# High incidence of microsatellite instability and loss of heterozygosity in three loci in breast cancer patients receiving chemotherapy: a prospective study

**DOI:** 10.1186/1471-2407-12-373

**Published:** 2012-08-28

**Authors:** Nasir Kamat, Mohammed A Khidhir, Mohammed Jaloudi, Sabir Hussain, Mouied M Alashari, Khaled H Al Qawasmeh, Ulf Rannug

**Affiliations:** 1 Department of Genetics, Microbiology and Toxicology, Stockholm University, Stockholm, Sweden; 2 Department of Genetics Research, Management of Natural Conservations, Abu Dhabi, UAE; 3 Department of Oncology and Hematology, Tawam Hospital, Abu Dhabi, UAE; 4 Department of Pathology, Tawam Hospital, Abu Dhabi, UAE

**Keywords:** Chemotherapy, Breast cancer, Genetic instability, Microsatellites, Mismatch repair, Loss of heterozygosity

## Abstract

**Background:**

The aim of the study was to evaluate potential chemotherapy-induced microsatellite instability, loss of heterozygosity, loss of expression in mismatch repair proteins and associations with clinical findings in breast cancer patients, especially resistance to chemotherapy and/or development of other tumors in the four years following chemotherapy treatment.

**Methods:**

A comprehensive study of chemotherapy-related effects with a follow-up period of 48 months post treatment was conducted. A total of 369 peripheral blood samples were collected from 123 de novo breast cancer patients. Microsatellite instability and loss of heterozygosity in five commonly used marker loci (including Tp53-Alu of the tumor suppressor gene TP53) were analyzed in blood samples. Sampling was conducted on three occasions; 4–5 weeks prior to the first chemotherapy session (pre-treatment), to serve as a baseline, followed by two consecutive draws at 12 weeks intervals from the first collection. Mismatch repair protein expression was evaluated in cancer tissues using immunohistochemistry for three mismatch-repair related proteins.

**Results:**

A total of 70.7% of the patients showed microsatellite instability for at least one locus, including 18.6% marked as high-positive and 52.1% as low-positive; 35.8% showed loss of heterozygosity in addition to microsatellite instability, while 29.3% exhibited microsatellite stability. The following incidence rates for microsatellite instability and loss of heterozygosity were detected: 39.1% positive for Tp53-Alu, 31.1% for locus Mfd41, and 25.3% for locus Mfd28. A higher occurrence of loss of heterozygosity was noted with alleles 399 and 404 of Tp53-Alu. The mismatch repair protein expression analysis showed that the chemotherapy caused a loss of 29.3% in hMLH1 expression, and 18.7% and 25.2% loss in hMSH2 and P53 expression, respectively. A strong correlation between low or deficient hMSH2 protein expression and occurrence of mismatch repair/loss of heterozygosity events in Mfd41, Tp53-Alu, and Mfd28 was evident. A significant association between mismatch repair/loss of heterozygosity and incidence of secondary tumors was also established.

**Conclusion:**

Our results suggest that microsatellite instability, loss of heterozygosity, and deficiency in mismatch repair may serve as early prognostic factors for potential chemotherapy-related side effects in breast cancer patients.

## Background

The rates of chemotherapy-related secondary cancers have increased considerably during the last two decades [1-8]. A variety of chemotherapy agents and combinations are used in oncology clinics, and side effects are inescapable. Genotoxic side effects in particular are believed to play a crucial and determinant role in treatment outcome for some patients [9,10]. Assessing changes at the genetic level in patients receiving chemotherapy is possible using several markers, and one of the most reliable choices is microsatellite markers [11,12].

Microsatellites or simple sequence repeats are tandem repetitive DNA consisting of arrays of one to five base pairs with a different number of repeat units. These DNA sequences are particularly prone to mutations via insertion–deletion loop formation during DNA synthesis, generating new allele lengths [13,14]. This microsatellite instability (MSI) and loss of heterozygosity (LOH) are the two aberrations known to be early steps in the tumorigenesis pathway [15,16]. Furthermore, MSI has been described as a replication error phenotype. Mismatch repair (MMR) pathways normally correct most of these replication errors, and microsatellite mutation rates are significantly elevated in the absence of MMR proteins, such as hMLH1, hMSH2, hMSH6, and hPMS2 [17-20].

Because systemic chemotherapy is an integral part of the treatment regimen in breast cancer, a large number of patients in both adjuvant and palliative settings show varied adverse effects that include phenotypic and/or genotoxic features, such as MSI and LOH [2,21-24]. Changes in the genomes of patients receiving chemotherapy, especially alkylating agents, can lead to many abnormal clinical phenotypes, such as a higher resistance to chemotherapy remedies and secondary cancers like acute myeloid leukemia and/or myelodysplasia, with an incidence rate of 1–5% [2,3]. MSI and/or LOH pathways work by accumulating mutations in genes responsible for tumorigenesis that are targets for mismatch-induced frameshift mutations e.g., transforming growth factor β1 receptor type II [25], insulin-like growth factor type II receptor [26], and the BAX gene [27]. In 1997, a National Cancer Institute (NCI) workshop developed the Bethesda guidelines, a set of clinical criteria to prompt MSI testing [28]. The conference recommended the use of a panel of five microsatellite sequences to assess instability. This panel consisted of two mononucleotide (Bat-25 and Bat-26) and three dinucleotide (D2S123, D5S346, and D17S250) repeat sequences. It was unanimously agreed that testing should compare DNA from tumor tissue and normal tissue, and classification of the results depends on the number of altered microsatellite sequences in the tumor relative to the normal tissue. If alteration is present in two or more of the five microsatellite sequences, the cancer is classified as high (MSI-H); if only one is mutated, it is classified as low (MSI-L); and if no changes are present among the five microsatellites, the tumor is considered microsatellite stable (MSS) [29]. The NCI panel of markers had been used frequently as a guideline for survival and molecular profiling in colorectal cancer and for screening patients for hereditary nonpolyposis colorectal carcinoma (HNPCC) [30,31]. Recently, high MSI detection was found to have clinical application in assisting the diagnosis of suspected HNPCC [32]. Many modifications of this panel have been adopted successfully in several studies. For instance, Bacher et al. [33] used a panel of five mononucleotide microsatellites (BAT-25,BAT-26, NR-21, NR-24 and MONO-27) and found it to be more sensitive and specific than the original NCI panel for screening colorectal cancer patients, while Fonseca et al. 2005 developed and used a set of markers (that includes BAT-26, BAT-40, MFD-28, MFD-41, TP53.PCR15.1, TP53ALU) specifically for screening breast cancer patients [30,33-35].

The five microsatellites (BAT-26, BAT-40, MFD-28, MFD-41, and TP53ALU) applied in the present study were also used by Fonseca and co-workers, although we analyzed a significantly larger and unique cohort compared to earlier studies [34,36]. Furthermore, the results presented are from a region with a high incidence of breast cancer, but at the same time for which epidemiological data are scanty [37,38]. In addition, an extended follow-up period of 48 months was included to see whether it was possible to match the detected MSI, LOH, and MMR expression to clinical findings, especially resistance to chemotherapy and/or development of other tumors in the four years following chemotherapy treatment. Collectively, such data could serve the purpose of establishing a solid link between chemotherapy and some of its negative consequences.

## Methods

### Study design and chemotherapy protocol

The 123 de novo breast cancer patients (ages 26–79 years) selected for this study had not received any previous chemotherapy. The chemotherapy regimen was as follows: 80.5% of the patients received 3–4 cycles of an FEC regimen, which consists of 5-fluorouracil, epirubicin, and cyclophosphamide (Cytoxan), and was administered at a low or high concentration (Table 1). In 46 patients, this regimen was followed by docetaxel. Our research conformed with the Helsinki declaration and local legislations and has been approved by Al Ain medical district human research ethics committee/ Faculty of Medicine and Health sciences, University of UAE under ethical permit No. AAMD/HREC 08/15.

**Table 1:** Chemotherapy regimen and positive cases detected

**Chemotherapy regimen**	**No. of patients**	**No. of MSI-H**	**No. of MSI-L-**	**Recurrence /2**^**nd**^**CA**
**High conc. FEC**^**a**^	**68**	**19**	**37**	**14**
5-Floururacil 500 mg/m^2^				
Epirubicin 90–100 mg/m^2^				
Cyclophosphamide 600 mg/m^2^				
**Low conc. FEC**^**a**^	**31**	**4**	**18**	**2**
5-Floururacil 300–400 mg/m^2^				
Epirubicin 50–75 mg/m^2^				
Cyclophosphamide 400 mg/m^2^				
**AC**^**b**^	**13**	**0**	**6**	**0**
Adriamycin (Doxorubicin) 60 mg/m^2^				
Cyclophosphamide 600 mg/m^2^				
**TC**^**c**^	**3**	**0**	**2**	**0**
Taxotere (Docetaxel) 75 mg/m^2^				
Cyclophosphamide 600 mg/m^2^				
**PT**^**d**^	**2**	**0**	**1**	**0**
Carboplatin (Paraplatin) AUC 6 mg/ml/min				
Taxotere 175 mg/m2				
**TA**^**e**^	**1**	**0**	**0**	**0**
Taxotere 90 mg/m^2^				
Avastin (Bevacizumab) 10 mg/kg				
**TH**^**f**^	**3**	**0**	**0**	**0**
Taxotere 90 mg/m^2^				
Herceptin (Trastuzumab) 4 mg/kg				
**LT**^**g**^	**1**	**0**	**0**	**0**
Lapatinib (Tykerb) 1500 mg po				
Taxotere 175 mg/m^2^				
**TE**^**h**^	**1**	**0**	**0**	**0**
Epirubicin 75 mg/m^2^				
Taxotere 200 mg/m^2^				
**Total**	**123**	**23**	**64**	**16**

Abbreviations: ^a^ = 5-Floururacil, Epirubicin and Cyclophosphomide, ^b^ = Adriamycin and Cyclophosphamide, ^c^ = Taxotere and Cyclophosphamide, ^d^ = Paraplatin and Taxotere, ^e^ = Taxotere and Avastin, ^f^ = Taxotere and Herceptin, ^g^ = Lapatinib and Taxotere, ^h^ = Epirubicin and Taxotere.

### Blood sample and cancer tissue collection

A total of 369 peripheral blood samples were collected from the patients. Sampling was conducted on three occasions starting from 4–5 weeks prior to the first chemotherapy session (pre-treatment), to serve as a baseline, and followed by two consecutive draws at 12-weeks intervals from the first collection. Twelve weeks interval represents the period required to complete a standard 3–4 cycles FEC regimen. The first post-treatment samples would demonstrate the presence of treatment-related MSI and LOH, and the second post-treatment samples would show the level of persistence of the first post-treatment findings. Simultaneously, 180 control samples were collected from 60 healthy individuals without any relevant reported symptoms, following the same sampling protocols.

In addition to blood sampling, 218 cancer tissues resected from the study patients were collected from the pathology department for MMR expression analyses. Among the collected breast cancer tissues, 54% were evaluated to be grade III, 30% grade II, 11% grade I, and 5% grade VI. Follow-up studies were performed for 36–48 months post-chemotherapy treatment. Patients were monitored for chemotherapy-related MSI and LOH, MMR expression and tumor recurrences and/or development of secondary tumors.

### DNA extraction and LOH and MSI analysis

Genomic DNA was extracted from whole blood using DNA isolation kit I, on a MagnaPure-LC extraction system (Roche, Germany).

Single and multiplex PCR reactions were conducted to amplify the five loci Mfd41 and Tp53-Alu on chromosome 17 and Bat-26, Bat-40, and Mfd28 on chromosomes 1, 2, and 10, respectively, using fluorescently labeled primers; the sequences have been described previously [39] and these markers were used by Fonseca et al. [34], for screening breast cancer patients. The PCR amplifications were performed according to Dietmaier and co-workers [39] with a slight modification of the cycling conditions. The specific primer information is given in Table 2. Amplification reactions were prepared in 10 μl reaction volumes of 1× Gold AmpliTaq Master Mix (Applied Biosystems, USA), with the addition of 80 ng purified genomic DNA and adjustment of the final concentration of primers to 0.4 μM. The following cycling conditions were applied: initial denaturation at 95°C for 5 minutes, followed by 31 cycles at 94°C for 1 minute, 55°C for 45 seconds, and 72°C for 50 seconds, with a final 40-minutes extension at 70°C. All runs included the use of an internal molecular weight control (LIZ 500 Genescan, Applied Biosystems). PCR products were loaded on an ABI 3130 genetic analyzer (Applied Biosystems, USA), and fragments were measured and compared using GeneMapper software Version 4. 

**Table 2:** Characteristics of microsatellite markers analyzed

**Name (locus)**	**Primer sequence (5’ to 3’)**	**Unit of repeats**	**PCR-Tm**^**c**^	**Dye**	**Size (bp)**
**TP53-Alu**	**F…GCA CTT TCC TCA ACT CTA CA**	**5**	**55°C**	**FAM**	**382-417**
	**R…AAC AGC TCC TTT AAT GGC AG**				
**Mfd41 (D17S261)**	**F…CAG GTT CTG TCA TAG GAC TA**	**2**	**55°C**	**NED**	**153-172**
	**R…TTC TGG AAA CCT ACT CCT GA**				
**Mfd28 (D10S89)**	**F…AAC ACT AGT GAC ATT ATT TTC**	**2**	**55°C**	**FAM**	**139-156**
	**R…AGC TAG GCC TGA AGG CTT CT**				
**Bat-40**	**F…ATT AAC TTC CTA CAC CAC AAC**	**1**	**55°C**	**VIC**	**116-132**
	**R…GTA GAG CAA GAC CAC CTT G**				
**Bat-26**	**F…TGA CTA CTT TTG ACT TCA GCC**	**1**	**55°C**	**FAM**	**112-127**
	**R…AAC CAT TCA ACA TTT TTA ACC C**				

GeneScan data counted a minimum peak detection limit of 50 relative fluorescent units using the local Southern size calling method. Alterations in the number of microsatellite alleles or allele distribution in the post-treatment samples compared to the pre-treatment samples were considered indicators of MSI [34,36,39]. In addition, when a peak height of one of the two heterozygote alleles was reduced by at least 35%, it was recorded as LOH [36]. The GeneMapper software is designed to calculate the LOH in two steps, first by calculating allele ratio for each sample by dividing the peak height of allele 1 by the peak height of allele 2 of the same sample, and second by calculating allelic imbalance by dividing the allele ratio of the pre-treatment sample by the allele ratio of the first post-treatment sample.

### MMR analysis

Cancer tissues sampled for initial pathological assessment have been used as pre-treatment specimens, while the surgically resected cancer tissues after chemotherapy regimen were utilized for post-treatment analysis. Tissues were embedded in paraffin blocks for MMR protein expression analyses. Healthy tissue from each patient was used as an internal control in addition to the standard controls provided by the manufacturer. In the staining procedure, the TP-125 HLX UltraVision Plus Anti-Polyvalent HRP detection system (Lab Vision, USA) was applied, using specific monoclonal antibodies for hMLH1, hMSH2, and P53 (Sigma Aldrich, Germany). Results were marked as positive when a 10% or higher proportion of cell nuclei stained positively.

### Statistical analyses

Confidence intervals were calculated at the 95% and 99% levels. Fisher’s exact test and Chi square were used for statistical analysis, with the SPSS statistical analysis package. In addition, an inter-rater reliability test using Cohen's kappa coefficient was used to measure correlation between the MSI and LOH results for the five markers and low MMR protein expression [40].

## Results

### Blood samples

Screening of the five microsatellite markers showed that 87 (70.7%) patients out of the total cohort of 123 breast cancer patients tested positive for at least one locus. These patients could be classified either as MSI-H due to MSI/LOH in two or more loci in each individual or as MSI-L (MSI/LOH in only one locus); 18.6% and 52.1% of patients were categorized in the first and second groups, respectively (Table 1). Among the positive cases, 50% exhibited LOH. The rest of the studied patients, 36 individuals (29.3%), tested negative for all markers and were reported as MSS. In terms of the 369 blood samples drawn for the study, we found that 28.5% displayed MSI (22.8% of first post-treatment and 5.7% of second post-treatment), and 23.8% samples showed LOH (11.9% in each post-treatment group).

**Figure 1:**
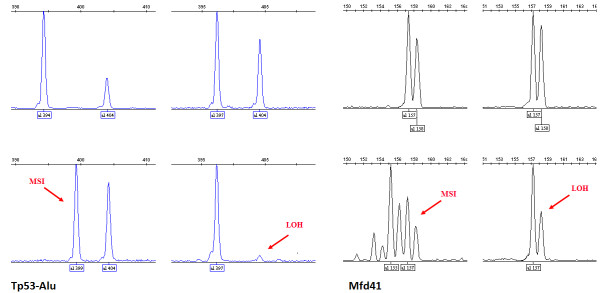
**Microsatellite instability and loss of heterozygosity in TP53-Alu (patient # 41 &103) and Mfd41 (patient # 72 & 56).** Upper panels represent normal genotypes, while the lower panels show examples of MSI and typical LOHs.

Comparing the induced changes in the individual microsatellites of the 87 patients, the following incidence rates of MSI and LOH were detected. The highest and lowest incidences were seen with Tp53-Alu (39.1%) and Bat-26 (4.6%), respectively, while Mfd41 and Mfd28 exhibited intermediate frequencies (Table 3). MSI was manifested in more than one way; either as additional peaks or as a novel allele within the size range of the marker, while a complete or partial loss of one of the two heterozygote alleles (35–100% loss of the original peak height) was recorded as an LOH event (Figure 1). The highest incidence of LOH was recorded in alleles 399 and 404 of microsatellite Tp53-Alu (Figure 1), which is located within the tumor suppressor gene TP53. Indeed, Tp53-Alu was the most informative among the five markers used in this study. Nevertheless, Mfd41 results approached the same level of instability despite the fact that it has fewer alleles (Table 3). In addition, Mfd41 showed MSI of alleles 157 and partial LOH of alleles 158 and 159 (Figure 1). No instability was detected in the microsatellite sequence Bat-40.

**Table 3:** Incidence rate of MSI and LOH, number of alleles isolated, and allelic imbalance noticed for each marker

**Marker**	**Chr./Locus**	**No of positive patients**	**Incidence of MSI / LOH**	**No. of alleles isolated**	**Allelic imbalance**
**TP53-Alu**	**17p13.1**	**34**	**39.08%**	**11**	**L 0.00 - 0.64**
					**U 1.52 - 2.61**
**Mfd41**	**17p12-11.1**	**27**	**31.03%**	**8**	**L 0.00 - 0.56**
					**U 1.37 - 2.82**
**Mfd28**	**10pter**	**22**	**25.28%**	**5**	**L 0.00 -0 .61**
					**U 1.42 - 5.83**
**Bat-26**	**2p**	**4**	**4.59%**	**3**	**L 0.30 - 0.52**
					**U 1.42 - 1.54**
**Bat-40**	**1p13.1**	**0**	**0%**	**4**	**L 0.73 - 0.79**
					**U 0.94 - 1.22**

L= lower allelic imbalance range.

U= upper allelic imbalance range.

#### Cancer tissues

**Figure 2:**
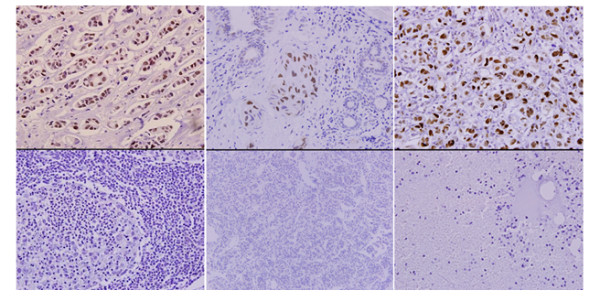
**Immunohistochemistry reactions for hMSH2, hMLH1 and P53 (Upper panel represent positive tissues and lower panel shows negative tissues)**.

The results from the immunohistochemistry analysis of MMR proteins showed that there was a significant difference (Fisher’s exact test) in MMR protein expression between cancer tissues sampled prior to chemotherapy and the resected tissues analyzed after the chemotherapy treatment. The treatment-dependent loss of protein expression was 29.3% (p < 0.0001) for hMLH1, and the corresponding loss for P53 and hMSH2 was 25.2% (p < 0.0001) and 18.7% (p = 0.003), respectively (Figure 2).

An average follow-up of 48 months showed that 13% of patients had a recurrent primary tumor and/or developed secondary tumors. All 16 of these patients were among those previously detected to have chemotherapy-related MSI/LOH in their first post-treatment sample, (11 patients [8.9%] MSI-H and 5 [4.1%] MSI-L). Recurrence of the primary disease was noticed in 8 patients, while secondary tumors were identified in 13 patients in lungs, brain, liver, bone marrow or blood. Fisher’s exact test results indicated a significant association between MSI/LOH and the incidence of secondary tumors (2-sided Fisher's exact = 0.014, and 1-sided Fisher's exact = 0.010).

### Association of changes with chemotherapy regimen

As Table 1 shows, patients who received the high-concentration FEC regimen were the majority among the recruited cohort (68 out of 123 patients); this group also represented the higher incidence of MSI-H (19 out of the 23 cases). After 48 months of follow-up, 14 of 16 patients experiencing recurrence were from this group. The group of patients who received the FEC regimen of lower concentrations had a higher incidence of MSI-L and less MSI-H and much less incidence of recurrence (Table 1). Some smaller groups who received different regimens had some MSI incidence but were insufficient in number of positive cases for statistical analysis.

### Other statistical analysis

As Table 4 shows, there was a significant (99% significance level) and strong correlation between low or deficient hMSH2 protein expression and the occurrence of MSI/LOH events in Mfd41, Tp53-Alu, and Mfd28. A strong correlation also emerged between LOH events in Tp53-Alu and low or deficient P53 expression (kappa = 0.595, P < 0.0001). No correlation was found between MSI/LOH events in Bat-26 and Bat-40 and low MMR expression.

**Table 4:** Correlation between MMR expression and MSI and LOH events

		**Mfd41**	**TP53-Alu**	**Mfd28**	**Bat-26**
**hMLH1**	**Kappa**	**−0.154**	**0.148**	**−0.241**	**−0.062**
	**p-value**	**0.080**	**0.101**	**0.005**	**0.191**
	**Significance level**	**ns**	**ns**	**	**ns**
**hMSH2**	**Kappa**	**0.822**	**0.744**	**0.375**	**−0.059**
	**p-value**	**< 0.0001**	**< 0.0001**	**< 0.0001**	**0.329**
	**Significance Level**	***	***	***	**ns**
**P53**	**Kappa**	**0.112**	**0.595**	**−0.121**	**0.000**
	**p-value**	**0.213**	**< 0.0001**	**0.168**	**0.992**
	**Significance Level**	**ns**	***	**ns**	**ns**

* kappa = measure of agreement between two tests.

## Discussion

Our findings clearly demonstrate a significant incidence of MSI and LOH in three out of the five markers screened and a strong correlation between MSI/LOH events and low expression of MMR proteins. As noted, both LOH and MSI are integral parts of the tumorigenesis process [15,16]. The incidences of chemotherapy-related MSI and LOH in post-treatment specimens, together with the follow-up findings that all cases of recurrent primary tumors and/or secondary tumors were encountered in patients diagnosed with MSI/LOH, led to an initial assumption that MSI and LOH play a pivotal role in determining treatment outcome and/or facilitate the development of secondary tumors. MSI, as mentioned above, is a replication error phenotype resulting from the reduced fidelity of the replication process [41]. Improperly functioning MMR machinery has been frequently implicated in the decreased fidelity or reduced proofreading efficiency in the replication apparatus as related to MSI [42,43]. Low fidelity of DNA replication, which results in accumulation of mutations, eventually generates the characteristic genetic instability phenotypes in precancerous and cancerous cells [44,45]. Research in this field was motivated in the first place by the findings that HNPCC is manifested with an elevated incidence of MSI in up to 85% of patients [46,47]. HNPCC patients have a high incidence of MSI, usually due to (a) silenced MMR gene(s), as in the case of hypermethylation of the hMLH1promoter [48]. It should be kept in mind that replication errors occur in repetitive DNA sequences at a higher frequency than in non-repetitive DNA sequences, making these repetitive sequences more error-prone in nature, and therefore particularly dependent on an efficient MMR [18,19].

The sequential accumulation of mismatched base pairs and/or LOH from multiple mutations may lead to deregulation of tumor suppressor genes such as TP53, VHL, FHIT, and Rb, which often are found to be inactivated in early precancerous and cancerous cells [49-51]. Our results showed that the occurrence of MSI and LOH was highest in the Tp53-Alu microsatellite among the markers screened**,** and it was also found to correlate with low or deficient expression of the hMSH2 and P53 proteins in the *de novo* breast cancer patients after chemotherapy. The patients included in this work are a population that has not been studied previously, to our knowledge. The detected incidences of MSI, LOH, and MMR are significant and may play a role in the development of secondary malignancies and/or more resistant phenotypes of cancer.

As an example, the higher incidence of MSI/LOH in alleles of Tp53-Alu (chromosome 17) may specifically induce the initiation of these consequences, which might be reasonable for the multi-task protein TP53, which plays a role in activating MMR in response to DNA damage [52]. LOH on chromosome 17 in the vicinity of the TP53 gene inactivates it as a tumor suppressor in many tumors [53], which may result in genetic instability, a characteristic phenomenon in malignant cells. In addition, the essential role played byTP53 in initiating programmed cell death (apoptosis) in response to irreparable damage is disrupted in many cancer cells through LOH [54-56]. An example of somatic mutations in tumor suppressor genes associated with a resistant cancer phenotype has been revealed in a recent study in which somatic mutation in the retinoblastoma gene was linked with resistance to anthracyclines/mitomycin in breast cancer patients [57].

Moreover, the MSI/LOH events in alleles of Mfd41 and Mfd28, which correlated with low expression of hMSH2 and hMLH1, respectively, may be an indication of a weakened efficiency of the MMR machinery in patients. The strong correlation indicates that the MSI/LOH incidence rate is related directly to the level of expression of MMR proteins. Similarly, MSI/LOH events in Mfd41 were correlated with low or deficient hMSH2 expression but not with P53 or hMLH1.These findings agree with the results from the study by [34] that low expression of hMSH2 correlated with MSI/LOH in Mfd41. In addition, we identified a similar correlation between low expression of hMSH2 and MSI/LOH in Tp53-Alu and Mfd28. On the other hand, our outcomes are not in concordance with their results of no correlation between hMLH1 or P53 expression and MSI/LOH events. We found a significant correlation between low expression of hMLH1 and P53 with MSI/LOH events in Mfd28 and Tp53-Alu, respectively.

In terms of the influence of dosing regimen, patients on the high-concentration FEC regimen--most of the cohort--had a greater number of MSI-H outcomes and were by far the most represented among the recurrences. The patients receiving the lower-concentration regimen had a much higher rate of MSI-L outcomes and far fewer cases of recurrence. These results provide indirect evidence of dose effect in chemotherapy-related side effects. However, some limitations and difficulties should be mentioned; for instance, we needed to repeat PCR runs and to do fine tuning for reaction conditions and fragment analysis in 32% of the patients to reduce stuttering and yield more clear results. Moreover, in twelve cases where patients have to move or discontinue their treatment in Tawam hospital, they were replaced by new participants and in five patients when the first tissue samples were processed by another hospital and reported to the pathology department in Tawam hospital the samples could not be successfully retrieved.

In conclusion, MSI/LOH events play a role in tumorigenesis as two of the genetic instability features in precancerous cells. Screening of breast cancer patients receiving chemotherapy for MSI and LOH can be of predictive value in assessing the level of chemotherapy-induced genetic instability and the possible development of secondary malignancies and/or resistant cancer phenotypes.

## Conclusions

Chemotherapy-induced genotoxic side effects especially MSI and LOH were detected in *de novo* breast cancer patients after 12 weeks of receiving their first treatment. During the four years follow-up period low MMR expression was noted, and correlated with clinical phenotypes such as secondary tumors or higher chemotherapy-resistant cancers. Of special interest was the higher incidence of LOH in two of the alleles of TP53-Alu, the microsatellite located within the tumor suppressor gene *TP53*. MSI/LOH of this microsatellite was strongly correlated with low or deficient MMR protein expression. A significant association between MSI/LOH and incidence of secondary tumors was also established in the study. Our results suggest that MSI, LOH, and MMR may serve as early prognostic factors for potential chemotherapy-related side effects in breast cancer patients.

## Abbreviations

FEC: Flourouracil-Epirubicin-Cyclophosphamide (Regimen); HNPCC: Hereditary Nonpolyposis Colorectal Carcinoma; hMLH1: Human mutL homolog 1; hMSH2: Human mutS homolog 2; LOH: Loss of heterozygosity; MMR: Mismatch repair; MSI: Microsatellite instability; MSS: Microsatellite stable.

## Competing interests

The authors have no competing interests.

## Authors’ contributions

NK study design, carried out the molecular genetic studies, and drafted the manuscript. MAK participated in the study design and reviewed written material. MMA carried out the immunohistochemistry assays. MJ participated in selection of study subjects and performed clinical follow up for the period of the study. SH participated in selection of study subjects and performed clinical follow up for the period of the study. KHAQ participated in coordination and follow up of the study effectively. UR study design, review and interpretation of results and final editing and approval of the manuscript for publishing. All authors read and approved the final manuscript.

## Pre-publication history

The pre-publication history for this paper can be accessed here:

http://www.biomedcentral.com/1471-2407/12/373/prepub

## Author details

^1^Department of Genetics, Microbiology and Toxicology, Stockholm University, Stockholm, Sweden. ^2^Department of Genetics Research, Management of Natural Conservations, Abu Dhabi, UAE. ^3^Department of Oncology and Hematology, Tawam Hospital, Abu Dhabi, UAE. ^4^Department of Pathology, Tawam Hospital, Abu Dhabi, UAE.
